# Neuroimaging Correlates of Cognitive Dysfunction in Adults with Multiple Sclerosis

**DOI:** 10.3390/brainsci11030346

**Published:** 2021-03-09

**Authors:** Maria Petracca, Giuseppe Pontillo, Marcello Moccia, Antonio Carotenuto, Sirio Cocozza, Roberta Lanzillo, Arturo Brunetti, Vincenzo Brescia Morra

**Affiliations:** 1 Department of Neurosciences, Reproductive and Odontostomatological Sciences, University of Naples “Federico II”, 80131 Naples, Italy; maria.petracca@unina.it (M.P.); moccia.marcello@gmail.com (M.M.); carotenuto.antonio87@gmail.com (A.C.); vincenzo.bresciamorra2@unina.it (V.B.M.); 2 Department of Advanced Biomedical Sciences, University of Naples “Federico II”, 80131 Naples, Italy; giuseppe.pon@gmail.com (G.P.); siriococozza@hotmail.it (S.C.); brunetti@unina.it (A.B.); 3 Department of Electrical Engineering and Information Technology, University of Naples “Federico II”, 80125 Naples, Italy

**Keywords:** cognitive dysfunction, neuroimaging, multiple sclerosis, magnetic resonance imaging, positron emission tomography, sodium, atrophy

## Abstract

Cognitive impairment is a frequent and meaningful symptom in multiple sclerosis (MS), caused by the accrual of brain structural damage only partially counteracted by effective functional reorganization. As both these aspects can be successfully investigated through the application of advanced neuroimaging, here, we offer an up-to-date overview of the latest findings on structural, functional and metabolic correlates of cognitive impairment in adults with MS, focusing on the mechanisms sustaining damage accrual and on the identification of useful imaging markers of cognitive decline.

## 1. Introduction

Cognitive impairment, which can be present from the very early or even preclinical stages of the disease [1,2], has been reported in 34 to 65% of adults with multiple sclerosis (MS) [3]. The domains more frequently affected are processing speed, sustained and selective attention, learning and episodic memory [4,5], with executive functions compromised in more advanced, progressive stages [6]. The development of cognitive impairment mirrors the high individual variability that characterizes the manifestation of physical disability in MS and results from neurodegeneration [7], disconnection of salient regions [8] and functional reorganization, which, to a certain degree, is able to counteract the clinical manifestation of structural damage [9] contributing, together with intellectual enrichment [10], to cognitive reserve [11]. Conventional imaging can only give a glimpse into these complex underlying mechanisms, while the application of advanced neuroimaging techniques offers a more exhaustive picture of the different correlates of cognitive impairment [12,13,14]. In this narrative review, we provide an overview of the structural, functional and metabolic correlates of cognitive impairment in adults with MS, focusing on the more recent findings achieved via advanced neuroimaging.

## 2. Structural Correlates of Cognitive Impairment

### 2.1. White Matter Damage

**Figure 1:**
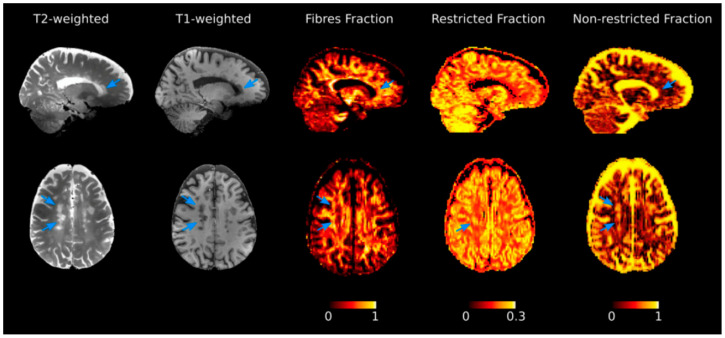
Diffusion basis spectrum imaging (DBSI) maps for the investigation of tissue properties. Selected T2- and T1-weighted images, together with DBSI-derived maps from a patient diagnosed with multiple sclerosis. Variations of each metric in normal appearing tissue in comparison with lesions (indicated by arrows) can be appreciated. The color bar expresses each DBSI-metric adimensional unit of measure. Reprinted from *Brain*, Vol. 144, Issue 1, P213–223, 12 February 2021. Schiavi S. et al. “Non-invasive quantification of inflammation, axonal and myelin injury in multiple sclerosis” [42]. Copyright: © 2021, with permission from Oxford University Press.

The role of white matter (WM) focal lesions as correlates of global cognitive impairment or deficit in specific cognitive functions has been widely explored in MS. Initial findings from cross-sectional studies [15,16] have been confirmed by longitudinal reports highlighting the role of short-term lesion load changes in predicting cognitive performance at follow-up [17,18,19], but evidence remains contradictory [16]. Although it is not possible to directly compare studies conducted with different designs in different populations, the overall emerging message is that demyelinating lesions play a minor role in comparison with atrophy and normal appearing tissue damage [20,21]. Mechanistically, demyelinating lesions impact on cognition is related to disruption of relevant WM tracts interconnecting key grey matter (GM) regions [22]. This is true not only for nonsocial (e.g., information processing speed, memory and executive functions) cognitive domains but also for social (i.e., emotion recognition, theory of mind and empathy) cognitive domains, as highlighted by a recent review interpreting the available findings in the context of a multiple disconnection syndrome [23]. Indeed, the presence of focal lesions translates in a disruption of the brain structural network [24] and results in density reduction in lesioned tracts and impaired function [25], with damage to long-range connections impacting brain network efficiency [26]. The effects of normal appearing WM (NAWM) disruption on cognition have been confirmed by different imaging techniques, from magnetization transfer (MT) and diffusion based to quantitative MR imaging [21,27,28,29,30,31,32,33], exploiting their sensitivity to different pathological aspects of the disease [34]. Parameters derived from diffusion tensor imaging (DTI), such as fractional anisotropy (FA), radial diffusivity (RD) and axial diffusivity (AD), correlate with histological measures of myelination as well as with axonal density [35,36]. For myelin assessment, magnetization transfer ratio (MTR) and the longitudinal relaxation rate (R1) represent more direct approaches, with R1 showing the highest sensitivity in comparison with other established myelin-sensitive MRI metrics [37]. More recently, diffusion-based models such as diffusion basis spectrum imaging (DBSI) and diffusion kurtosis imaging (DKI) have been developed to characterize water diffusion properties associated with a wider range of pathological correlates [38,39] (Figure 1), with neurite orientation dispersion and density imaging (NODDI) seemingly able to capture pre-symptomatic axonal degeneration in animal models of neurodegeneration [40]. Applying DBSI and DKI, fiber density and axonal damage within NAWM have been confirmed as significant correlates of cognitive function [41,42]. Notwithstanding their limitations (i.e., modelling of crossing fibers, modelling of focal lesion effects on structural connectivity, discrimination of pathological from physiological variations in fiber orientation and dispersion) [43,44,45], advanced dMRI models, reflecting the intricacy of the central nervous system (CNS) microstructure, represent a valuable tool not only for the exploration of damage accrual but also neuroplastic mechanisms of functional recovery in MS [46,47].

### 2.2. Gray Matter Damage

**Figure 2:**
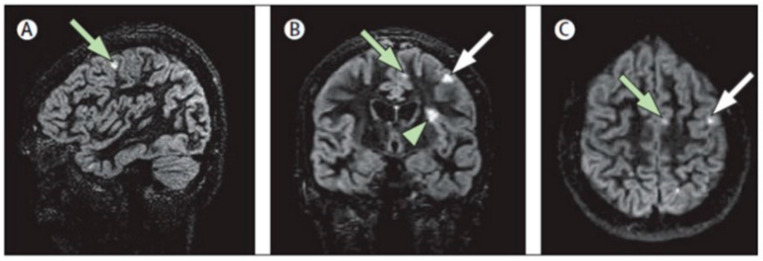
Multiplanar representation of single-slab 3D double-inversion recovery images of a patient with MS. (**A**) Sagittal view of a juxtacortical lesion (arrow) in the frontal vertex. (**B**) Coronal orientation: same wedge-shaped juxtacortical lesion (white arrow), as well as a mixed grey matter–white matter (type I) lesion (arrowhead) near the frontal operculum and a smaller juxtacortical lesion frontomedially (green arrow). (**C**) Same two juxtacortical lesions as shown in (**A**,**B**), in the axial orientation. Reprinted from *The Lancet Neurology*, Vol. 7, Issue 9, P841–851, 1 September 2008 [60]. Copyright: © 2021, with permission from Elsevier.

GM lesions (both cortical and subcortical) represent an early and frequent phenomenon in MS [48], which has emerged as a specific and clinically relevant disease biomarker. This has led to the inclusion of the cortical lesion location in the diagnostic criteria [49] and to the gradual integration of dedicated MRI sequences for the in vivo assessment of cortical lesions (e.g., double-inversion recovery, DIR, phase-sensitive inversion recovery, PSIR or high-resolution 3D magnetization prepared rapid acquisition with gradient echo, MPRAGE) [50,51,52,53] into both diagnostic and follow-up imaging guidelines [54,55] (Figure 2). Indeed, the number and volume of GM lesions have been consistently linked not only to physical disability, but also to cognitive impairment [56,57], independently from GM volume loss [58], adding to the sole quantification of WM lesion burden [59] and with possible region-related effects, such as the suggested association between hippocampal lesions and visuospatial memory impairment [57].

Besides focal GM lesions, a wide spectrum of microstructural modifications is known to occur also in the normal appearing GM (NAGM) of MS patients, going beyond (and associated with) GM atrophy [60]. The study of NAGM abnormalities in vivo, made possible by advanced MRI techniques including magnetization transfer imaging (MTI) [61], quantitative relaxometry [62], quantitative susceptibility mapping (QSM) [63] and diffusion MRI [64], has provided valuable insights into MS pathophysiology and has proved worthwhile in terms of clinical correlations, even if it is not always clear to what extent these correlations are driven by GM atrophy [60]. In particular, globally reduced MT ratio in the NAGM, considered as an index of demyelination, has been related to cognitive impairment in MS patients [65,66]. In accordance with this finding, T1 relaxometry studies demonstrated an association between cognitive impairment and higher mean values and skewness of GM T1-relaxation time, both reflecting demyelination, mainly in the thalamus and fronto-temporal cortices [67,68,69]. Furthermore, diffusion MRI studies, leveraging DTI [70] or alternative diffusion models [71], demonstrated that altered NAGM integrity (resulting from a combination of demyelination, axonal damage, gliosis, dendritic stripping) contributes to the explanation of cognitive deficit in MS patients, with abnormal diffusion metrics in specific GM regions associated with worse performance in distinct cognitive functions [70,71,72]. Finally, studies exploiting quantitative iron-sensitive MRI techniques showed mixed results, with cognitive performance negatively correlating with both magnetic susceptibility (reflecting iron content) in the globus pallidus [72] and with T2*-relaxation time (a measure inversely correlated with both iron and myelin content) in selective cortical regions [73].

### 2.3. Atrophy

Brain atrophy and cognitive impairment are both detectable from the earliest stages of MS [74,75]. Different studies have pointed to GM atrophy as a key determinant of cognitive impairment [3]. In particular, cortical GM atrophy could be involved in the development of specific attentional deficits [76], from the early stages of MS [31]. In a 5-year longitudinal study, reduced cortical GM volume at baseline was associated with the development of cognitive decline, suggesting a leading role of cortical atrophy [77].

Although simple MRI proxy of brain atrophy are useful surrogate marker of global disability [78], improvements in MRI post-processing have granted the possibility to segment different components of white and grey matter [74,79], allowing refinement of associations with cognitive features. In particular, thalamus and other deep GM structural changes play an important role in the development of the full spectrum of cognitive impairment in MS [3,31,80,81,82], including attention-processing speed, executive function, fluency, visuo-spatial working memory and verbal memory [80,83]. Thalamic atrophy, induced by the interplay of local and remote pathologic processes [84,85], has been associated with cognitive impairment from the early stages of MS and could further enhance cognitive changes in patients with more pronounced WM lesion load [15]. During the course of the disease, the progression of cognitive changes mirrors deep GM volume changes, suggesting a direct association [77]. Similarly, cerebellar atrophy has been associated with cognitive changes in attention-processing speed, visuo-spatial working memory and verbal memory [20]. Besides, hippocampal atrophy looks more specifically related to visuo-spatial working memory and verbal memory deficits [32]. 

In conclusion, structural changes in the GM have been associated with cognitive impairment in MS, with the involvement of multiple neuropsychological domains and brain areas, including the thalamus, cerebellum and hippocampus.

### 2.4. Network Modifications

In recent years, looking at the brain as a complex system consisting of many neural elements whose interconnection at different scales of space and time underlies clinical–cognitive functioning, pathophysiological models of brain disorders have shifted from an emphasis on characterizing pathology in specific regions to understanding disturbances at the level of networks, defined by connections and their topology [86].

**Figure 3:**
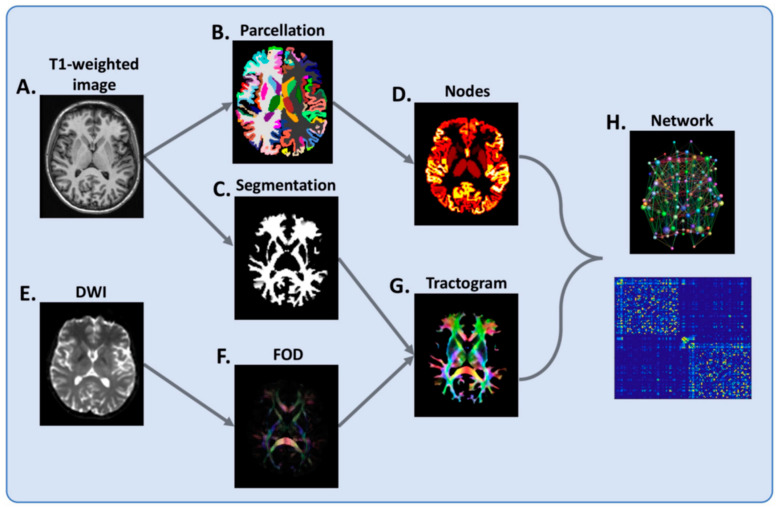
Flowchart of brain network reconstruction. For each subject, (**A**) T1-weighted image is segmented into grey matter (**B**) and white matter (**C**). The grey matter segmentation is parcellated into cortical and deep grey matter regions (**B**), which serve as network nodes (**D**) in the subsequent network-based analysis. From a diffusion-weighted image (DWI) (**E**), voxel-wise fiber orientation distribution (FOD) (**F**) is estimated and whole-brain tractography undertaken (**G**), with the white matter segmentation (**C**) used to prevent this from spilling into grey matter. Finally, nodes and tractogram are modelled into a network (**H**). Connections are weighted by the sum of the pairwise streamline weights. Reprinted from *JNNP*, Vol. 90, Issue 2, 01 February 2019 [91]. Copyright: © 2021, with permission from BMJ Publishing Group Ltd.

MRI provides the means to assess the macro-scale architecture of brain connectivity, characterized as the anatomical links between different GM regions estimated with diffusion MRI tractography, by the similarity in GM morphological features (i.e., cortical thickness) derived from structural MRI or by the statistical dependence of GM regions’ signal time courses in functional MRI [87]. While network-based analyses conducted so far offered mixed results, which are not easily interpretable in the light of clinical outcomes, a number of diffusion MRI studies seem to point towards a reduction in topological efficiency (a measure of network integration) in MS patients’ structural connectome [88,89], which is best explained by the disruption of long-range WM tracts [26] and the disconnection of main hubs such as the thalamus and nodes of the default-mode network (DMN) [90], and underlies cognitive dysfunction [91,92] (Figure 3).

Accordingly, MS has been described as a disconnection syndrome, with “network collapse” suggested as a putative mechanism through which cumulative structural brain pathology ultimately leads to clinical–cognitive disability [9,93].

Furthermore, studies exploring the patterns of anatomical covariance between GM regions demonstrated not only that GM atrophy in MS patients occurs according to distinct spatial patterns, which partially explains the variability of cognitive outcomes [94], but also that structural damage in MS tends to disrupt the coordinated patterns of morphological homology between GM regions, leading to a more random GM network organization, which correlates with worse cognitive functioning independently of GM atrophy [95,96].

## 3. Functional and Metabolic Correlates

### 3.1. Functional MRI

Functional MRI (fMRI) is an advanced imaging technique based on the detection and evaluation of blood oxygen level-dependent (BOLD) changes that occurs during neuronal activity. Indeed, neuronal activation leads to an increase in metabolic requirements from brain parenchyma, which is achieved by increasing the arterial blood flow. Nevertheless, this increase is not coupled to a similar capillary extraction of oxygen, which leads, therefore, to a relative decrease in deoxyhemoglobin concentrations that is possible to detect via MRI as an index of neuronal activity [97].

With a great effort of synthesis, fMRI studies can be divided in task and rest-related experiments. While task fMRI studies measure BOLD changes after an active execution of any performance (e.g., a motor task, a verbal fluency test, etc.), the latter approach evaluates the interactions occurring between different brain regions without the execution of any specific task and, for this reason, goes under the name of resting-state fMRI (RS-fMRI). This approach allows for the demonstration of the presence of preferential functional connectivity (FC) between cerebral structures, which results in organized and stable and reliable networks [97].

Although in the past years several studies have investigated possible changes in cerebral functions by means of task-related fMRI experiments in MS patients [98,99,100,101,102,103], a great contribution to our understanding of functional brain damage in MS, with particular reference to cognition, comes from RS-fMRI studies. Among all the networks that have been studied in MS, a central role has emerged for DMN modifications. Indeed, FC changes affecting some of the nodes of this network have been reported in MS, both in terms of an increased [104] and a decreased [105,106] FC, as well as changes in local regional homogeneity [107] and variability [108].

**Figure 4:**
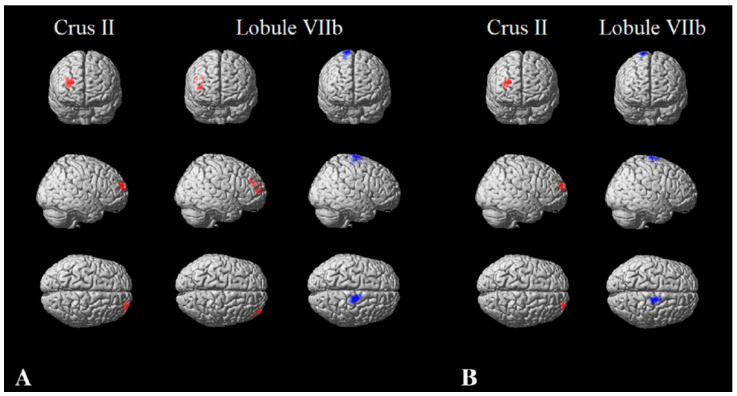
Cerebellar functional connectivity in multiple sclerosis. Cerebellar functional connectivity modification in patients with multiple sclerosis compared to controls, without taking into account (**A**) and after controlling (**B**) for cerebellar structural damage. Clusters of significant functional connectivity decrease are shown in red, while clusters of significant functional connectivity increase are presented in blue, superimposed on a standard 3D rendering of a brain volume in the Montreal Neurological Institute space. Reprinted from *JOON*, Vol. 265, Issue 10, P2260–2266, October 2018 [111]. Copyright: © 2021, with permission from Springer Nature.

In particular, an increased FC of the posterior cingulate gyrus has been reported in clinically isolated syndrome, which appears to be lost in relapsing–remitting patients [104]. On the other hand, a decrease in the FC of the anterior cingulate cortex, which is an anterior hub of the DMN, has been found in relapsing–remitting patients and, in particular, in patients showing a relatively preserved cognitive function [105]. These results suggest that cognitive impairment might contribute in a different way to MS-related DMN changes in relapsing–remitting MS, at least at the level of this anterior hub of the DMN [105]. To further support this finding, a reduction in the anterior cingulate cortex FC was also found when primary-progressive patients where compared to subjects with secondary-progressive MS, with activity reduction more pronounced in patients with cognitive impairment, therefore suggesting the possible involvement of this area as one of the factor causing the accrual of cognitive deficits in progressive MS patients [106]. It is noteworthy to mention that FC changes have been identified not only in supratentorial areas, but also the infratentorial compartment. In particular, cerebellar FC changes have been reported in pediatric [109], relapsing–remitting [110] and progressive MS [111] patients, showing some degree of correlation with cognitive scores, further supporting the role of cerebellum in cognition [20,112,113,114,115,116] (Figure 4). As per the nature of the reported FC changes, they seem to play a compensatory role to structural damage in the early disease stages, until damage accrual determines a network collapse leading to manifest cognitive deficit [9]. Again, this holds true also for the less explored social cognition domains, where fMRI studies [117,118] showed regional hyperactivation in individuals without social cognition deficits, providing evidence of adaptative functional reorganization, with hypoactivation on fMRI and/or social cognition deficits [119] identifiable only at later disease stages.

### 3.2. Positron Emission Tomography

**Figure 5:**
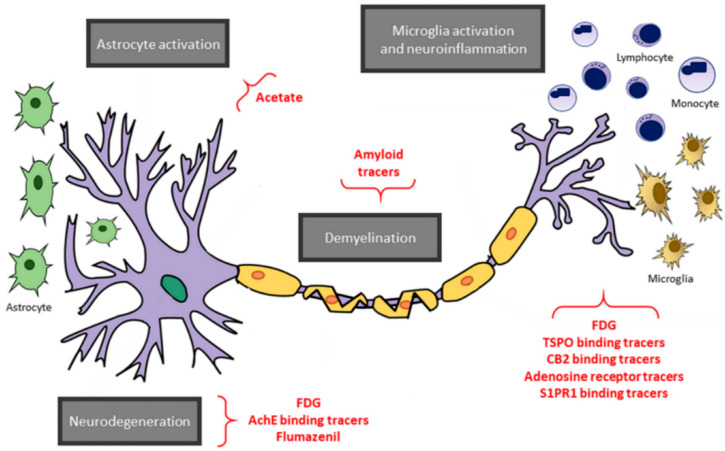
An overview of positron emission tomography (PET) targets and tracers. A schematic representation of the main targets of PET imaging in multiple sclerosis (gray boxes), along with the respective tracers (in red). Reprinted from *EJNMMI Radiopharmacy* and *Chemistry* Vol. 4, Issue 1, P6, 8 April 2019 [120]. Copyright: © 2021, with permission from Springer Open.

Positron emission tomography (PET) is a quantitative non-invasive imaging technique that allows to measures in vivo the distribution of an exogenous radionuclide targeting specific cellular molecules. Therefore, it is well placed to study biological substrates underpinning MS pathology. So far, three pathological processes occurring in MS have been explored through PET: inflammation, demyelination/remyelination and axonal degeneration (Figure 5).

Inflammation was mostly evaluated by measuring microglia activation targeting the peripheral benzodiazepine receptor (PBR), also known as TSPO. To date, TSPO-binding [^11^C]PK11195 ligand has been widely used in MS. [^11^C]PK11195 PET studies have shown an increased tracer distribution in the NAWM, T2-, T1- and Gadolinium-enhanced cortical and subcortical GM in MS patients compared with healthy controls [121,122,123,124,125,126]. Interestingly, at the very early stages of the disease, patients already display an increased extent of microglia activation throughout NAWM, which predicts disease course over the follow-up [127,128]. Additionally, it has been demonstrated that increased neuroinflammation in the thalamus is linked with cortical atrophy [129]. In the same study, authors associated cognitive impairment with neuroinflammation in the cortex, deep GM and NAWM [129]. Since both cortical atrophy and a widespread inflammatory status were already associated with cognitive impairment, of great interest would be the investigation of the temporal dynamic linking atrophy, inflammation and cognitive impairment, in order to disentangle the contribution of neurodegeneration and neuroinflammation to cognition [30,56,130].

Neurodegeneration could also be assessed in MS through PET imaging by measuring either GABAA receptors, through [^18^F]flumazenil or two-deoxy-2-[fluorine-18]fluoro-d-glucose ([^18^F]FDG) (Figure 5). The latter measures the metabolic rate of glucose utilization (CMRglu), and hence, it is a marker of presynaptic neuronal function [130]. Using [^18^F]flumazenil PET imaging, Freeman et al. demonstrated that MS patients show an overall decreased tracer binding in the GM, which was associated with cognitive impairment [131]. Notably, such decreased [^18^F]flumazenil uptake was not associated with atrophy detected through conventional MRI imaging. Therefore, [^18^F]flumazenil PET imaging seems to be able to detect GM pathology even before MRI markers of neurodegeneration are evident. Similarly, PET imaging studies using [^18^F]FDG showed that MS patients have a reduced CMRglu in the GM, which worsens over the disease course [132]. Hypometabolism in deep GM is associated with cognitive impairment especially in thalamus [133], outlining the central role of this structure in cognitive function. However, despite these findings, [^18^F] FDG lacks specificity, and its application in MS is limited due to high background binding within the brain.

Finally, also, demyelination/remyelination assessment in MS has received growing interest. Lesion load as well as lesion location and the extent of tissue damage within demyelinating lesions are strongly associated with cognitive impairment and could also predict cognitive worsening overtime [30]. First used tracers for myelin were amyloid-β protein targeting ligands (Figure 5). These stilbenes and benzothiazole derivatives have a very flat structure, interacting with the secondary structure of myelin basic protein similarly to the way they interact with amyloid [134,135]. Actually, myelin basic protein has a particular two-dimensional secondary structure, acting as a hinge between lipid bilayers. In this structure, stilbene and benzothiazole derivatives are trapped, and hence, when lipid bilayers are damaged, a lower uptake is detectable. The most used tracers to assess myelination are the Carbon-11-labeled Pittsburgh compound B ([^11^C]PIB), [^18^F]florbetaben and [^18^F]florbetapir [136,137]. To date, only ^18^F-Florbetaben uptake was associated to the extent of cognitive impairment [138]. Therefore, further studies integrating MRI structural analysis and PET data are highly needed in order to understand whether PET may reveal myelin damage beyond what can be detected through standard MRI, thus contributing to the exploration of cognitive dysfunction in MS. Amyloid PET imaging might also be used to differentiate MS-related cognitive decline from other neurodegenerative diseases associated with amyloid deposition such as Alzheimer’ s disease. To date, no studies have been performed to explore this possibility. As matter of fact, while amyloid deposition for Alzheimer’ s disease occurs throughout GM, and hence, analyses focus on this compartment, amyloid PET imaging in MS does not measure amyloid deposition, as it focuses on WM compartment, and the binding is not specific as for amyloid protein. A multi-parametric study demonstrated the association between Florbetapir PET uptake in NAWM and cerebrospinal fluid β-amyloid [139]. However, in this case, β-amyloid reflected the extent of neuronal damages and not protein accumulation. A study performing a between-group comparison using healthy volunteers, MS patients with and without cognitive impairment and patients with β-amyloid accumulation-related neurodegenerative diseases may shed light on the possible role of amyloid PET imaging as tool to solve differential diagnosis issues.

In conclusion, despite its promising results and potential applications, clinical application of PET imaging is still hampered by the high costs, the need for skilled operators both for data acquisition and analysis, the low PET resolution and the need for development of specific tracers. However, the introduction of hybrid PET/MRI scans may be useful to reduce costs and acquisition time, allowing the simultaneous acquisition of PET and MRI imaging with the advantage of evaluating with a higher extent of specificity biological processes associated with MS pathology.

### 3.3. Magnetic Resonance Spectroscopy

Magnetic resonance spectroscopy (MRS) is an imaging technique allowing the measurement of metabolites in the brain when these are present in relatively high concentrations. Differently from MRI, mainly relying on proton (^1^H), MRS can rely on a variety of nuclei. However, ^1^H-MRS is the most used methodology when it turns to neurological disorders, as it allows us to evaluate a large number of molecules such as the N-acetylaspartate (NAA), glutamate (Glu) and glutamine (Gln), choline (Cho), creatine (Cr) and myoinositol (mI). Each of this molecule reflects ongoing pathological processes.

NAA is an amino acid derivative synthesized in neurons and transported down over the axons. It is considered a specific marker of neurons, axons and dendrites integrity [140,141]. In MS, NAA can be measured either as absolute value or normalized for intra-voxel Cr concentration (NAA/Cr). NAA levels were shown to be lower in acute lesion with concentrations only partly recovering over the follow-up, being lower than healthy controls’ WM [142,143]. Similarly, NAA concentration also progressively reduces in NAWM from the very early stages, with the lowest concentrations observed in progressive phenotypes [144,145]. Data concerning NAA concentration in GM are still conflicting and deserve to be further investigated. All in all, NAA may be considered as a marker of axonal loss and/or metabolic dysfunction. NAA concentration in NAWM, and not in GM, has been associated with attention functioning in MS [146]. As NAA levels are markers of axonal loss, one may argue that a closer association between cognition and NAA levels would have been expected in GM rather than in NAWM. Perhaps, it could be hypothesized that axonal transection due to demyelination over WM fiber bundles revealed by NAA levels may precede axonal loss occurring in GM. Longitudinal analyses may shed further light on this topic.

Differently from NAA, Glu and Gln are considered as markers of inflammation, and increased Glu and Gln levels are associated with neurotoxicity, as they are excitatory neurotransmitters [147]. In MS, a widespread increase in Glu and Gln levels has been observed in WM lesions, NAWM and GM both in the relapsing and progressive phenotypes [148,149,150,151]. Interestingly, Glu levels in NAWM are also associated to brain atrophy, disability accrual and cognitive impairment [147,149], thus mirroring results obtained through TSPO PET imaging [129]. While Glu is an excitatory neurotransmitter, gamma-aminobutyric acid (GABA) is the major inhibitory neurotransmitter in the brain. The assessment of GABA levels in MS is still debated. Available findings suggest that a reduction in GABA in GM is associated with motor function in MS [152]. Interestingly, such a reduction might be related over time to a reactive increase in functional connectivity that can minimize clinical worsening [153]. To date no association has been reported between GABA levels and cognition.

Cho signal reflects cell membrane metabolism and elevated Cho concentrations represent a high cell membrane turnover manifesting during demyelination, re-myelination, inflammation and gliosis in MS patients [154]. Several studies reported increased Cho levels in NAWM of relapsing MS patients and such an increase seems to precede the appearance of gadolinium enhancing lesions [143,155]. However, in progressive MS patients, data are still conflicting, and no conclusion can be drawn [155,156]. Cho in cortical GM was associated with the PASAT3, a cognitive test assessing sustained attention [146]. Therefore, although axonal transection over fiber bundles may occur and underpins cognitive impairment, also a higher membrane turn-over likely contributes to this dysfunction, probably mirroring ongoing damages in the axonal soma.

Cr and mI levels were shown to be associated to both energy metabolism and gliosis [140]. Overall, Cr and mI levels increased in WM lesions, NAWM and GM in MS patients compared with healthy controls [152,157,158].

In conclusion, MRS is a promising technique that allows the quantification of metabolites concentrations reflecting MS pathological processes. Nevertheless, it has technical limitations, such as the very low spatial resolution and high spectra sensitivity to variations in B0 and B1 magnetic fields, that still need to be addressed before clinical application can be implemented.

### 3.4. Sodium MRI

**Figure 6:**
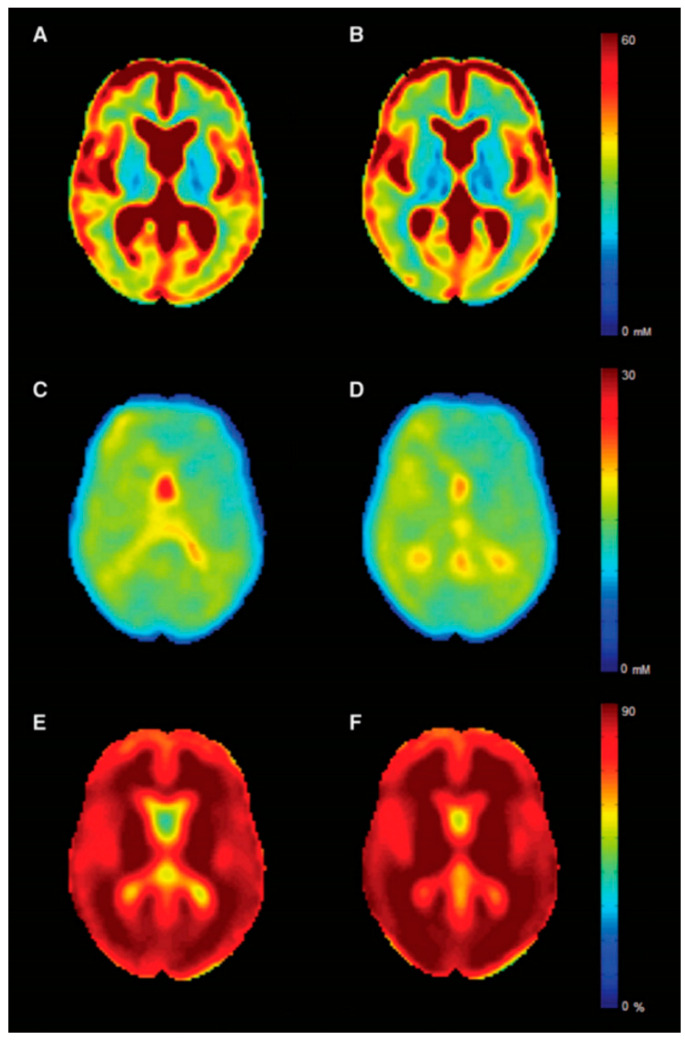
Sodium maps. Mean sodium MRI maps for multiple sclerosis patients (left column) and controls (right column). In the first row is displayed the total sodium concentration (**A**,**B**), while in the middle and last rows, the intracellular sodium concentration (**C**,**D**) and the intracellular sodium volume fraction (indirect measure of extracellular sodium concentration) (**E**,**F**) are shown. Reprinted from *Brain*, Vol. 139, Issue 3, P 795–806, 20 January 2016 [167]. Copyright: © 2021] with permission from Oxford University Press.

Sodium imaging allows the estimation of brain total sodium concentration (TSC), which reflects intra- and extracellular sodium concentration [157] (Figure 6). TSC increase in MS, related to neuro-axonal metabolic dysfunction [158] and/or expansion of the extracellular space secondary to neuro-axonal loss or edema [159,160], has been reported by several independent groups [157,161,162,163,164,165,166]. Recently, TSC increase in the cortex of MS patients has shown an independent and better association with cognitive impairment than GM atrophy [161,164]. Although this might suggest a role for TSC as marker of neuronal dysfunction rather than an indirect measure of cellular loss, future studies exploring selectively the relation between cognition and intracellular sodium concentration, which more closely captures changes in cellular metabolism related to mitochondrial and ion channels dysfunction [157], are warranted to confirm this hypothesis.

## 4. Conclusions

Over recent years, the development of new MRI sequences and modelling approaches has allowed the characterization of many structural and functional correlates of cognitive impairment in MS, providing new tools for disease monitoring and identifying new potential therapeutic targets. Although many questions remain unanswered, the value of advanced neuroimaging as an investigative tool of pathological changes in vivo remains undisputed, and future developments in this field will steadily add to our understanding of cognitive involvement in MS.

## Abbreviations

[^11^C]PIB = carbon-11-labeled Pittsburgh compound B; [^18^F]FDG = two-deoxy-2-[fluorine-18]fluoro-d-glucose; AD = axial diffusivity; BOLD = blood oxygen level-dependent; Cho = choline; CNS = central nervous system; CMRglu = metabolic rate of glucose utilization; Cr = creatine; DBSI = diffusion basis spectrum imaging; DIR = double-inversion recovery; DMN = default-mode network; DKI = diffusion kurtosis imaging; DTI = diffusion tensor imaging; FA = fractional anisotropy; FC = functional connectivity; fMRI = functional MRI; GABA = gamma-aminobutyric acid; Glu = glutamate; Gln = glutamine; GM = grey matter; mI = myoinositol; MPRAGE = magnetization prepared rapid acquisition with gradient echo; MRS = magnetic resonance spectroscopy; MS = multiple sclerosis; MT = magnetization transfer; MTI = magnetization transfer imaging; MTR = magnetization transfer ratio; NAA = N-acetylaspartate; NAGM = normal appearing grey matter; NAWM = normal appearing white matter; NODDI = neurite orientation dispersion and density imaging; PBR = peripheral benzodiazepine receptor PET = positron emission tomography; PSIR = phase-sensitive inversion recovery; QSM = quantitative susceptibility mapping; R1 = longitudinal relaxation rate; RD = radial diffusivity; RS-fMRI = Resting-State fMRI; TSC = total sodium concentration; WM = white matter.
